# Busulphan-Cyclophosphamide Cause Endothelial Injury, Remodeling of Resistance Arteries and Enhanced Expression of Endothelial Nitric Oxide Synthase

**DOI:** 10.1371/journal.pone.0030897

**Published:** 2012-01-27

**Authors:** Sulaiman Al-Hashmi, Piet J. M. Boels, Fahad Zadjali, Behnam Sadeghi, Johan Sällström, Kjell Hultenby, Zuzana Hassan, Anders Arner, Moustapha Hassan

**Affiliations:** 1 Experimental Cancer Medicine (ECM), Department of Laboratory Medicine, Karolinska Institutet, Stockholm, Sweden; 2 3Ph_S Biomedical, Stockholm, Sweden; 3 Division Genetic Physiology, Department of Physiology and Pharmacology, Karolinska Institutet, Stockholm, Sweden; 4 Department of Molecular Medicine and Surgery (MMK), CMM, Karolinska Institutet, Stockholm, Sweden; 5 EMIL, Department of Laboratory Medicine, Karolinska Institutet, Stockholm, Sweden; 6 Clinincal Research Center, Karolinska University Hospital-Huddinge, Stockholm, Sweden; City of Hope National Medical Center and Beckman Research Institute, United States of America

## Abstract

Stem cell transplantation (SCT) is a curative treatment for malignant and non malignant diseases. However, transplantation-related complications including cardiovascular disease deteriorate the clinical outcome and quality of life. We have investigated the acute effects of conditioning regimen on the pharmacology, physiology and structure of large elastic arteries and small resistance-sized arteries in a SCT mouse model. Mesenteric resistance arteries and aorta were dissected from Balb/c mice conditioned with busulphan (Bu) and cyclophosphamide (Cy). *In vitro* isometric force development and pharmacology, in combination with RT-PCR, Western blotting and electron microscopy were used to study vascular properties. Compared with controls, mesenteric resistance arteries from the Bu-Cy group had larger internal circumference, showed enhanced endothelium mediated relaxation and increased expression of endothelial nitric oxide synthase (eNOS). Bu-Cy treated animals had lower mean blood pressure and signs of endothelial injury. Aortas of treated animals had a higher reactivity to noradrenaline. We conclude that short-term consequences of Bu-Cy treatment divergently affect large and small arteries of the cardiovascular system. The increased noradrenaline reactivity of large elastic arteries was not associated with increased blood pressure at rest. Instead, Bu-Cy treatment lowered blood pressure via augmented microvascular endothelial dependent relaxation, increased expression of vascular eNOS and remodeling toward a larger lumen. The changes in the properties of resistance arteries can be associated with direct effects of the compounds on vascular wall or possibly indirectly induced via altered translational activity associated with the reduced hematocrit and shear stress. This study contributes to understanding the mechanisms that underlie the early effects of conditioning regimen on resistance arteries and may help in designing further investigations to understand the late effects on vascular system.

## Introduction

Stem cell transplantation (SCT) is an important treatment for several malignant disorders including leukemia and solid tumors, as well as for non-malignant conditions such as metabolic and genetic diseases. The number of stem cell-transplanted patients is constantly increasing due to the broader applicability and ameliorated clinical outcome. SCT requires an intensive preparative conditioning regimen consisting of total body irradiation (TBI), chemotherapy, or a combination of both [1], [2], [3]. Despite a continuous improvement of SCT, several complications such as sinusoidal obstructive syndrome (SOS), graft versus host disease (GVHD), cardiac toxicity and treatment-related mortality are still major limiting factors. These factors are important in the determination of long term outcomes f SCT [4]. Although cardiac toxicity associated with SCT is a rare event, it is important in pediatric patients [5]. The cardiovascular events, including cardiac toxicity, heart failure and hypertension have been reported after systemic anticancer treatment [6] and after SCT with a frequency of 1–9% [5], [7], [8], [9], [10]. Nevertheless, the mechanisms underlying these complications have not been fully clarified. Several factors such as the conditioning regimen, infections and alterations in the immune system have been recently addressed and related to late cardiovascular problems [11], [12]. Injury to the vascular system may lead to fatal organ dysfunction involving the cardiovascular [9], [13] or respiratory systems [14].

Although cardiovascular complications have been reported mainly after allogeneic hematopoietic SCT, several reports have shown arterial dysfunction following autologous SCT, with a high incidence shortly after the transplantation [15], [16]. The high frequency of cardiovascular complications might indicate that the type and intensity of the conditioning regimen may play a role in their pathophysiology. In mouse models, the late complications appear to be less frequent in the syngeneic compared to the allogeneic settings [14].

Conditioning regimen prior to SCT aims to provide a space for the transplanted cells through myeloablation as well as to suppress the recipient's immune system in order to avoid rejection. About one half of the patients undergoing SCT are conditioned with chemotherapy without irradiation. Conventional anticancer chemotherapy has been correlated to serious complications such as cardiac infarction [17], [18], pulmonary arterial hypertension [19] and increased mortality [20]. During conditioning regimen, the patients are treated with higher doses of cytostatics compared to conventional chemotherapy. Busulphan (Bu) and cyclophosphamide (Cy) are alkylating agents commonly used in conditioning regimen prior to SCT [21], [22]. Cy is also used in many cancer treatment protocols [23] and in low doses in the treatment of several autoimmune diseases [24], [25]. Treatment with Cy has been related to cardiac toxicity and other types of tissue damage, e.g., hemorrhagic cystitis [26], [27]. Soon after the introduction of Cy in conditioning regimen, the onset of cardiotoxicity was reported by several authors [28], [29]. Moreover, several investigations have shown a positive correlation between the dose of Cy and severity of cardio toxicity [30]. The symptoms usually appear 10 to 20 years after SCT in patients with long term survival [31], but cardiac failure has been reported also within weeks of Cy exposure [32]. Bu, on the other hand, has not been associated with vascular toxicity. Nevertheless, it has been suggested that Bu can be a possible cause for pericardial fibrosis [33].

Since vascular alterations such as the damage of endothelial cells [34] or smooth muscles might occur long before the clinical manifestations of toxicity, it is difficult to establish the causative relationship between the treatment and cardiovascular side effects in human. Reports on cardiovascular toxicities after SCT in humans entail mainly case reports and retrospective patient studies, including post-mortem examinations. Thus, other treatments given concomitantly with chemotherapy, inheritance for cardiovascular diseases, life style or diet have to be considered. To our knowledge there are no systematic studies investigating the early onset or mechanisms underlying the cardiovascular damage that might occur during or soon after conditioning regimen using chemotherapy prior to SCT.

In this study, we assessed the early effects of Bu-Cy conditioning regimen prior to SCT on the vascular system in a mouse model using clinically relevant doses and conditions. Following the *in vivo* treatment with Bu-Cy, the effect on arterial microscopic, biochemical and reactivity properties was examined *in vitro* and we report significant changes in mechanical properties and in endothelial relaxant function of resistance arteries.

## Materials and Methods

### Animals and treatment

Female Balb/c mice (8–12 weeks old) were purchased from Scanbur, Sollentuna, Sweden. The animals were allowed to acclimatize for 1–2 weeks before the start of the experiments. Animals were kept in individual ventilated cages and fed standard food and water *ad libitum* in the pathogen-free part of the local animal house under controlled conditions. Air was filtered using HEPA filters; humidity, temparature and light/dark cycles were maintained at 55%±5%, 21±2°C and 12/12 h, respectively. All experiments were approved by Stockholm South Ethics Committee and conformed to the Swedish laws and European regulations on animal welfare (Approval S 57-08, S 183-10).

Busulphan (Bu, Sigma-Aldrich, Stockholm, Sweden), 20 mg/kg body weight, was injected intraperitoneally once daily for four consecutive days. After Bu administration, the animals received intraperitoneal injections of Cyclophosphamide (Cy, Sigma-Aldrich), 100 mg/kg body weight, once daily for two consecutive days, according to a previously published protocol [35]. Care was taken to inject caudally and contra-laterally to the side from which the small mesenteric arteries were taken (upper left quadrant of the abdomen). Control animals underwent the same injection procedure with PBS (phosphate buffered saline). The animals were sacrificed by cervical dislocation five days after the last injection and their body weight was recorded. The heart, aorta and mesentery were harvested in ice-cold Ca^2+^-free physiological salt solution (Ca^2+^-free PSS) and further dissected within 3 hours as described below. The heart was trimmed of non-cardiac tissues and structures and the wet weights (left and right ventricles) were registered. The dry weight of the hearts was also assessed after immersing the hearts in liquid nitrogen and thereafter vacuum drying overnight.

### Vessel isolation and mounting for isometric *in vitro* force registration

Mechanical properties and *in vitro* pharmacological reactivity were examined in ring-formed preparations of the thoracic aorta and the mesenteric microvasculature. Intestinal resistance arteries of the 2^nd^ and 3^rd^ branching order (running respectively perpendicular to or semi-parallel with the surface of the intestine) were used in the present experiments. All vessels were carefully freed of adhering fat, connective tissue and the accompanying vein.

A similar procedure was followed for dissection of ring preparations from the distal part of the thoracic aorta. The segment length of the mesenteric artery and aortic preparations was approximately 2 mm. Two stainless steel wires (diameter 40 µm) were inserted into the lumen of the mesenteric artery preparations, taking care not to overstretch the vessel longitudinally or extensively scrape the luminal side of the preparation. The stainless steel wires were then mounted parallel onto two specimen holders, one attached to a force transducer and the other to a one-dimensional micrometer-calibrated vernier.

The aorta rings were mounted on two parallel pins (0.2 mm diameter) in 5 mL organ baths of a Multi Wire Myograph system (DMT A/S, Aarhus, Denmark) essentially as previously described [36]. Dissection and mounting was performed in ice-cold Ca^2+^-free PSS (composition, see below) and was finished within 3 hours after animal sacrifice.

The Ca^2+^ free physiological salt solution contained in mM: NaCl 119, KCl 4.7, MgCl_2_, 1.2, KH_2_PO_4_, 1.2, NaHCO_3_, 25, glucose 11, Na_2_EDTA 0.03. The solutions were continuously gassed with 95%O_2_/5%CO_2_ giving a pH of 7.4 at 37°C.

### Determination of length-force relationship

**Figure 1:**
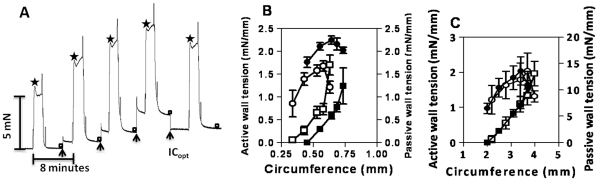
Circumference-tension relationships in control and Bu-Cy treated animals. (**A**) Original recording of force responses of a mesenteric artery from a Bu-Cy treated mouse. The vessel circumference was changed stepwise (**arrows**) between contractions induced by high-K^+^. Maximum active force at the peak of contraction (**Stars**) and passive force at the end of the relaxation period (**squares**) immediately before the next circumference step were determined. When the vessel had been stretched to a circumference above the optimal (IC_opt_), it was returned to IC_opt_ for further pharmacological experimentation. (**B** and **C**) show mean values of passive (squares) and active (circles) tension plotted against vessel circumference in resistance arteries (**B**; controls n = 4; Bu-Cy: n = 5) and aorta (**C**; controls n = 3; Bu-Cy: n = 6). Open and filled symbols show data of controls and Bu-Cy animals, respectively.

After mounting, all preparations were stretched to slack circumference (largest circumference whereby no passive force is manifest, reference slack internal circumference, IC_ref_) by careful adjustment of the vernier. Thereafter the preparations were equilibrated in Ca^2+^-containing PSS (2.5 mM CaCl_2_ added) for at least 30 minutes. The arteries were then activated every 8 min for 60 seconds with a high-K^+^ solution (high-K^+^ PSS, Ca^2+^-containing PSS with isotonic replacement of Na^+^ with 125 mM K^+^). The high K^+^-induced contraction was relaxed by re-application of pre-warmed and pre-oxygenated Ca-PSS between contractions. For mesenteric arteries, active force was recorded at the peak of contraction (within 15 s after activation). For the aorta, active force was recorded 60 s after activation since no clearly discernable initial peak was obtained. First, two contractions were obtained at IC_ref_. Active force was calculated by subtracting force at peak or at 60 s from the passive force obtained just before the activation period (see below). Passive tension was recorded prior to activation with high K^+^ and prior to the step – increase of circumference. This procedure (60 s activation, 5 min relaxation, stretch, 2 min relaxation) was repeated until the active force was no longer increasing after which the circumference was brought back to its previous optimal value (IC_opt_), (cf. also original record in Figure 1A). All preparations were then allowed to equilibrate for at least 30 min before further experimentation. Active and passive wall tension values were calculated from respective force values and the segment length at IC_opt_ (determined using a microscope with an ocular scale).

### Assessment of contractile and relaxant responses

The thromboxane A2 agonist U46619 (1×10^−5^ M), noradrenaline (1×10^−10^–1×10^−4^ M) (Sigma-Aldrich Sweden AB, Stockholm, Sweden) were applied to resting preparations cumulatively with log-unit increases of concentration. The maximum active force at each concentration was determined and normalized to the maximal high-K^+^ induced tension recorded for each preparation.

Acetylcholine (1×10^−10^–1×10^−4^ M), sodium nitroprusside (1×10^−10^–1×10^−4^ M) and forskolin (1×10^−10^–1×10^−5^ M) (Sigma-Aldrich) induced relaxations were initiated from a stable contraction induced by noradrenaline at a concentration of 1–30 µM, giving a stable initial tension level. The relaxant compounds were applied cumulatively with log-unit step increases in concentration. The extent of relaxation was expressed relative to the stable tension recorded before the vasodilators were added. In each experiment the basal force level in the fully relaxed state was recorded at the end of the experiment in the presence of 1 mM sodium nitroprusside and 0.2 mM papaverine (Sigma-Aldrich) in Ca^2+^ free PSS.

### Blood pressure

The mice were anaesthetized with isoflurane (2.6%; Univentor 400, AgnThos, Stockholm, Sweden) and placed on a servo-controlled heating pad maintaining body temperature at 37.5°C. Blood pressure was measured with a fiberoptic transducer (Samba 420/360, Samba sensors AB, Västra Frölunda, Sweden) inserted in the left carotid artery. After 15 minutes of stabilization, the pressure was continuously sampled for 10 minutes for later analysis in Chart v4.2 (AD Instruments Ltd, Oxford, UK). The augmented pressure was calculated as the systolic pressure minus the pressure at the augmentation point [37].

### Expression of the endothelial nitric oxide synthase

The mRNA expression was determined using RT-PCR. Total RNA was isolated from 3–5 mg of frozen mesenteric artery and aorta using the RNeasy Mini Kit (Qiagen, Valencia, CA, USA) according to the manufacturer's protocol. Total RNA (62 ng from mesenteric artery and 180 ng from aorta) was reverse-transcribed using the iSCRIPT™ cDNA synthesis kit (Bio-Rad, CA, USA). cDNA samples were amplified using 2× SYBR Green PCR Master Mix (Bio-Rad) at optimal concentrations (10 nmol/L) of primers in a total reaction volume of 20 µL under the conditions recommended by the manufacturer. Expression levels of genes were normalized to that of ribosomal RNA S18 to control for input gene. Samples were assayed in duplicate and expression profiles were generated using the comparative Ct method implemented in the Applied Biosystems 7500 Real-Time PCR System. The following primers were used (5′to3′): eNOS F: CCTTCCGCTACCAGCCAGA, eNOS R: CAGAGATCTTCACTGCATTGGCTA, S18 F: CGCGGTTCTATTTTGTTGGT and S18 R: AGTCGGCATCGTTTATGGTC. These results were confirmed by running the final product of the RT-PCR in 2.5% agarose gel.

For Western blotting analysis, 3–5 mg of arterial tissue was homogenized in 100 µL ice-cold buffer containing: 50 mM Tris.HCl (pH 7.5), 150 mM NaCl, 0.5% NP-40, 5 mM EDTA, 1 mM Na_3_VO_4_, 20 mM NaF and protease inhibitors cocktail (Roche, Mannheim, Germany), 1 mM dithiothreitol (DTT) and 1 mM phenyl-methylsulfonyl fluoride (PMSF). Homogenates were cleared by centrifugation (13,000 rpm; 15 min, 4°C) and the protein contents of the supernatant were determined using Bio-Rad Protein Assay (Bio-Rad Laboratories, CA, USA). Samples were prepared in 4× NuPAGE LDS sample buffer and 10× NuPAGE reducing agent (Invitrogen) and heated for 10 min at 80°C before electrophoresis. Five and 30 µg of proteins from mesenteric artery and aorta, respectively, were separated on 10% SDS-PAGE followed by transfer to a PVDF membrane. Membranes were blocked for 1 hour at room temperature in TBST containing 5% non-fat dry milk or BSA, followed by incubation with eNOS or β-Actin antibodies. Membranes were washed with TBST buffer (0.01% Tween-20) followed by incubation with HRP-IgG antibodies. Membrane blots were then exposed to ECL detection reagents (SuperSignal West Pico Chemiluminescent Substrate; Pierce; Biotechnology, Thermo Scientific, USA) and visualized using x-ray films. Band intensities were quantified by using Quantity One software (Bio-Rad. Laboratories). All antibodies were obtained from Santa Cruz Biotechnology (Santa Cruz, CA, USA).

### Transmission electron microscopy (TEM)

Mesenteric vessels were dissected as above and small pieces were fixed in 2.5% glutaraldehyde+1% paraformaldehyde in 0.1 M phosphate buffer, pH 7.4 at room temperature for 30 min and stored in the fixative at 4°C. Specimens were rinsed in 0.1 M phosphate buffer, postfixed in 2% osmium tetroxide in 0.1 M phosphate buffer, pH 7.4 at 4°C for 2 hours, dehydrated in ethanol followed by acetone and embedded in LX-112 (Ladd, Burlington, Vermont, USA). Semithin sections were cut and stained with toludine blue and used for light microscopic analysis. Ultrathin section (approximately 40–50 nm) were cut and contrasted with uranyl acetate followed by lead citrate and examined in a Tecnai 12 Spirit Bio TWIN transmission electron microscope (Fei Company, Eindhoven, The Netherlands) at 100 kV. Digital images were taken by using a Veleta camera (Olympus Soft Imaging Solutions, Münster, Germany) [38].

### Statistical analysis

Statistical analysis and curve fitting was performed using SPSS v16, Sigmaplot and GraphPad Prism. Student's *t*-test was used for two-group comparisons. All values are presented as mean ± SEM (Standard Error of the Mean), and with corresponding n values. To analyze the concentration-dependence of the vascular preparations to vasoconstrictor or vasodilator compounds, a hyperbolic (Hill) equation (Equation 1.) was fitted to the tension (y) and concentration (x) data (y = *M*×x*^h^*/(x*^h^*+*EC_50_^h^*)), where *M* is the extrapolated maximal tension at saturating concentration, *h* the Hill coefficient and *EC_50_* the concentration giving half-maximal response.

## Results

### Animal and heart weights

The Bu-Cy treated mice had a significant reduction in the body weight compared to control group (Table 1). However, no change in heart weight was observed between treated and non treated animals. To assess whether the water content of the cardiac tissue was altered, we examined the dry/wet weight of hearts in the both groups. No difference was found between treated and untreated groups.

**Table 1:** Body and heart weights of Bu-Cy treated and control animals.

	Body weight (g) start	Body weight (g) end	Heart weight (wet; mg)	Heart weight (dry; mg)	Heart weight (dry/wet)	R.V./L.V.
**Control**	22.2±1.1 (n = 7)	22.4±0.8 (n = 7)	81.5±2.1 (n = 13)	19.3±0.5 (n = 13)	0.237±0.002 (n = 13)	0.305±0.01 (n = 6)
**Bu-Cy**	22.5±0.5 (n = 13)	19.6±0.6 (n = 13)	79.5±2.3 (n = 13)	18.4±0.5 (n = 13)	0.232±0.002 (n = 13)	0.307±0.006 (n = 7)
**Significance**	n.s.	*p*<0.001	n.s.	n.s.	n.s.	n.s.

Body weights were determined at the start and end of treatment. Heart weights were determined at the end of treatment.

*P*: significance; n.s.: not significant; R.V.: right cardiac ventricle; L.V.: left cardiac ventricle.

### Circumference-tension relationships

The initial segment lengths were similar in the control and Bu-Cy vessels for the mesenteric artery (control: 1.9±0.1, n = 8; Bu-Cy: 1.7±0.1 mm, n = 9) and for the aorta (control: 1.3±0.1, n = 12; Bu-Cy: 1.4±0.1 mm, n = 19). The slack circumference of the relaxed vessels in the Ca^2+^-free solution (IC_ref_) was significantly larger for the Bu-Cy mesenteric arteries (control: 0.39±0.02, n = 16; Bu-Cy: 0.45±0.02 mm, n = 22, *P*<0.05). No difference was found in the aorta (control: 1.66±0.04, n = 8; Bu-Cy: 1.67±0.04 mm, n = 20).

Bu-Cy treatment increased the slack circumference of the mesenteric arteries but not of the aorta. Increased slack circumference was also reflected in an increased circumference at which active isometric force was maximal. The amplitude of the peak of the high K^+^-induced contraction was also larger in mesenteric arteries of Bu-Cy treated animals compared to control.

Figure 1 (A) shows an original record of an experiment determining the circumference-tension relationship in a mesenteric artery from a Bu-Cy treated animal. Similar recordings were performed on controls and on the aorta preparations. Figure 1 (B and C) show the summarized circumference-tension data from mesenteric arteries and aorta. Both the active and passive circumference-tension relationships of the mesenteric arteries from the Bu-Cy treated group were shifted towards larger circumferences and exhibited an increased maximal active tension. The relationships of the aorta preparations were similar in the two groups. Table 2 shows the optimal internal circumference (IC_opt_), the passive tension and the active tension at IC_opt_. The Bu-Cy mesenteric arteries had significantly increased IC_opt_ and maximal active tension, whereas no significant differences were found in the aorta. To analyze the passive elastic properties of the mesenteric arteries we extrapolated the relationship between tension and internal circumference (IC) to zero passive tension and determined the reference slack internal circumference (IC_ref_). The relationship between passive tension (PT) and IC/IC_ref_ is non-linear with a clearly higher stiffness (i.e., the steepness of the relationship between IC/IC_ref_ and PT) in the Bu-Cy groups. The IC/IC_ref_ at the optimal length for active force was significantly (*P*<0.001) lower in the Bu-Cy group compared to control group (Bu-Cy: 1.52±0.03, n = 6 and controls: 1.93±0.06, n = 4).

**Table 2:** Circumference-tension data of mesenteric arteries and aorta of Bu-Cy treated and control animals.

	Mesenteric artery			Aorta		
	IC_opt_ (mm)	PT (mN/mm)	AT (mN/mm)	IC_o_ (mm)	PT (mN/mm)	AT (mN/mm)
**Control**	0.579±0.019 (4)	0.73±0.12 (4)	1.68±0.11 (4)	3.68±0.21 (3)	9.02±1.87 (3)	2.03±0.51 (3)
**Bu-Cy treatment**	0.675±0.014 (5)	0.66±0.11 (5)	2.22±0.08 (5)	3.40±0.10 (6)	7.18±0.75 (6)	2.04±0.40 (6)
**Significance**	*p*<0.01	n.s.	*p<0.01*	n.s.	n.s.	n.s.

Optimal circumference for active force development (IC_opt_); passive wall tension (PT) and active wall tension (AT) at IC_opt_, were determined from circumference force relationships (cf. Figure 1). Values are the means ± SEM for (n) animals.

*P*: significance;

### Pharmacological reactivity

The circumference-tension data showed that passive tension at optimal circumference (IC_opt_) was similar in the control and Bu-Cy vessels, both for mesenteric arteries and aorta. We therefore stretched the preparations to passive tensions close to that at IC_opt_ for examination of effects of contractile and relaxant agonists. We normalized the contractile responses to the high-K^+^ tension, determined for each preparation.

**Figure 2:**
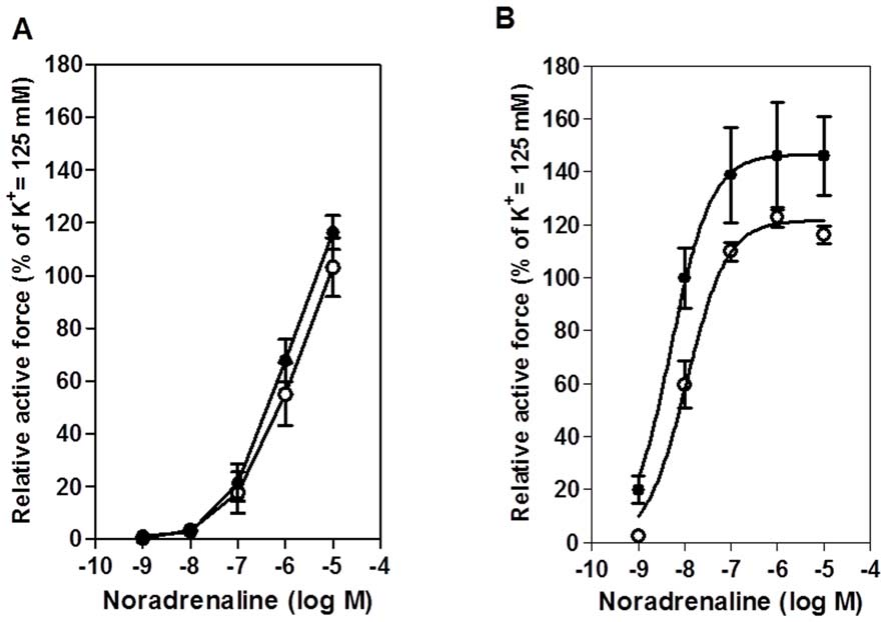
Effect of noradrenalin on relative active force (tension) in mesenteric arteries and aortas of Bu-Cy treated and control animals. Noradrenalin concentration–tension relationships for mesenteric arteries (**A**; n = 6 in each group) and aorta (**B**; n = 5 in each group). Vessels from control (open circle) and Bu-Cy treated (filled circle) animals. A hyperbolic equation (see Results) was fitted to mean values in **B** (Control: *M* = 119%, *EC_50_* = 7.99 −log(M), *h* = 1.3; Bu-Cy: M = 146%, *EC_50_* = 8.30 −log(M), *h* = 1.1).

Figure 2 shows noradrenaline concentration-force relationships of mesenteric arteries (Figure 2A) and aortas (Figure 2B) of control and Bu-Cy animals. The tension values were related to the high-K^+^ response. Within the concentration-range employed, noradrenalin active responses failed to plateau at higher concentrations, a phenomenon which, by contrast, was clearly observable in aortas.

However, the responses were clearly similar in the control and Bu-Cy groups, excluding major alterations in the contractile adrenoceptor signaling. For the aorta, the Bu-Cy vessels exhibited enhanced responses to noradrenaline with increased contraction amplitude of the responses at each concentration.

The *EC_50_* value was significantly lower in the Bu-Cy aorta preparations compared to control group (Bu-Cy: 8.30±0.08, n = 5; controls: 7.97±0.11, n = 5; −log (M), *P*<0.05). However, no difference was found between the groups with regard to *h* value (Bu-Cy: 1.2±0.1, n = 5; controls: 1.3±0.1, n = 5). The *M* values were slightly larger in the Bu-Cy group, but not significantly different (Bu-Cy: 145±18, n = 5; controls: 120±3; n = 5). In summary, the sensitivity to noradrenaline was unchanged in the mesenteric arteries and increased in the aorta.

To examine the effect of relaxant agonists, vessels were precontracted with U46619 (1 µM, mesenteric arteries) or noradrenaline (1 µM, aorta). The maximal initial active tension, relative to the high-K^+^ response was 1.20±0.06 (n = 11) and 1.21±0.11 (n = 6) in Bu-Cy and control resistance arteries, respectively and 1.52±0.18 (n = 6) and 1.48±0.16 (n = 3) in Bu-Cy and control aortas, respectively. In all preparations, active tension declined from this initial peak to a stable plateau.

**Figure 3:**
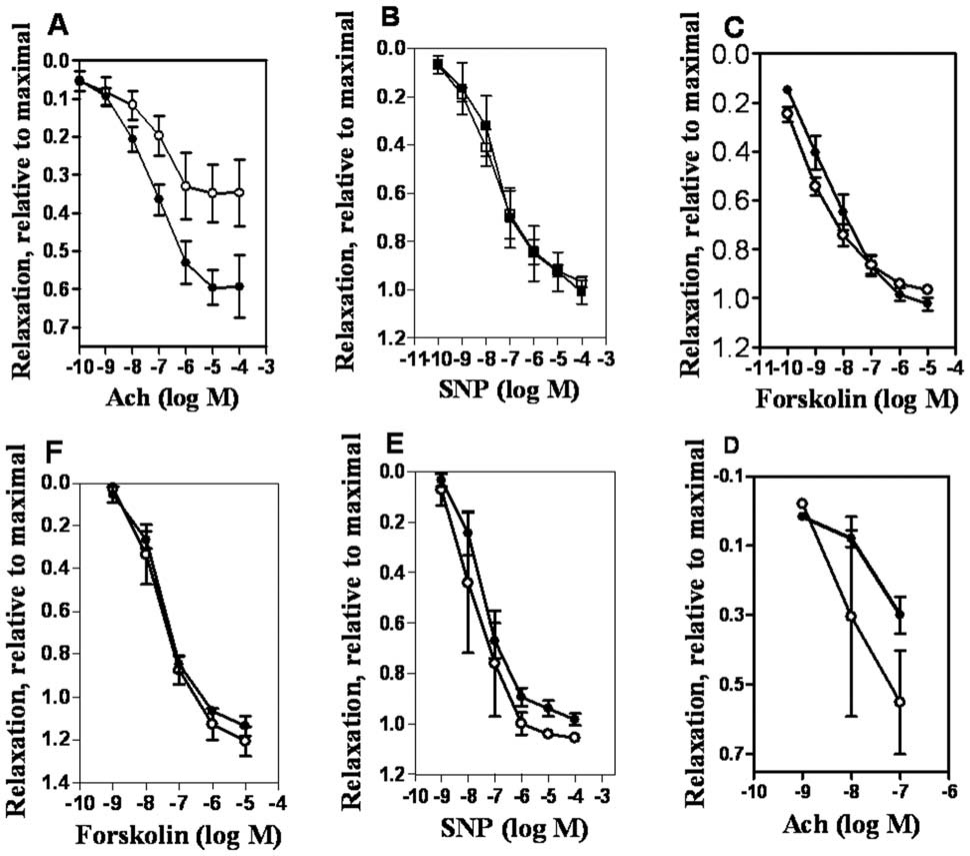
Relaxant responses to acetylcholine, sodium nitroprusside, and forskolin in resistance arteries and aorta of control and Bu-Cy treated animals. Relaxant responses to acetylcholine (Ach; **A** and **D**), sodium nitroprusside (SNP; **B** and **E**), and forskolin (**C** and **F**) in resistance arteries (**A–C**) and aorta (**D–F**) of control (○) and Bu-Cy treated (•) vessels, n = 3–9 in each group. A hyperbolic equation was fitted to the extent of relaxation in Panel **A** (Control: *M* = 0.38, *EC_50_* = 7.31 −log(M), *h* = 0.4; Bu-Cy: M = 0.63. *EC_50_* = 7.37 −log (M), *h* = 0.45). In all other diagrams, data are connected with straight lines. Extent of relaxation is related to maximum force induced by U46619 (mesenteric resistance artery) or noradrenalin (aorta), prior to addition of the relaxant agonist.

Next, we examined the relaxant responses to acetylcholine (endothelium-dependent relaxation), sodium nitroprusside (activating relaxant NO dependent pathways directly) and forskolin (activating cAMP dependent relaxation directly) in order to examine if this difference was due to changes in cellular smooth muscle cellular signaling or in the general ability to relax. Significant and rapid relaxations were recorded in both resistance arteries and aorta preparations in response to all these agonists (Figure 3). The resistance arteries of Bu-Cy treated animals relaxed to a significantly larger extent compared with the controls in response to acetylcholine (Figure 3A). The *M* values would correspond to the maximal extent of relaxation at saturating acetylcholine concentration. The *M* values in the Bu-Cy treated group (0.65±0.4, n = 7) were significantly (*P*<0.05) larger than those of the control vessels (0.40±0.11, n = 7). The *EC_50_* and *h* values for acetylcholine did not differ between the groups (*EC_50_* Bu-Cy: 7.2±0.3, n = 7; control: 7.2±0.3, n = 7, −log (M); *h* Bu-Cy: 0.53±0.07, n = 7; controls: 0.47±0.09, n = 6). To examine if the altered relaxation to acetylcholine was due to changes in the smooth muscle cells, we relaxed vessels with sodium nitroprusside (Figure 3B). No difference in the extent of relaxation or in the sensitivity to sodium nitroprusside was observed. Responses to forskolin were not different between the control and Bu-Cy treated mesenteric arteries (Figure 3C). Similar experiments were performed on the aorta preparations. In both control and Bu-Cy aortas, acetylcholine gave less prominent relaxations at doses above 10^−7^ M possibly reflecting activation of a contractile process. As seen in Figure 3D, E and F the relaxant responses to acetylcholine, sodium nitroprusside and forskolin were similar in the control and Bu-Cy groups. In summary, these results show that Bu-Cy treatment enhanced endothelium mediated relaxation in mesenteric resistance arteries but did not change relaxation properties of the aorta.

### Tissue expression of endothelial nitric oxide synthase

**Figure 4:**
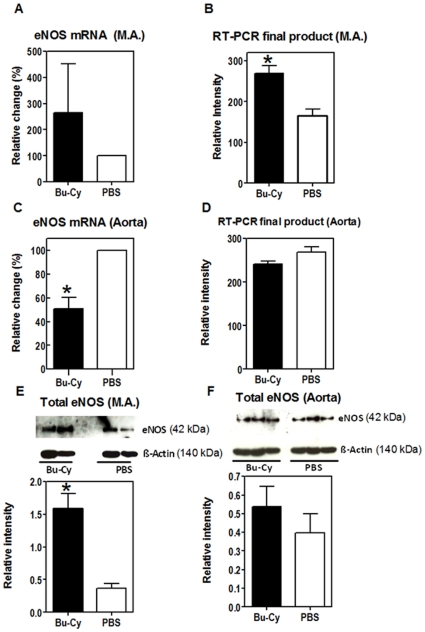
eNOS mRNA and protein expressions in mesenteric resistance arteries and aorta of control and Bu-Cy treated animals. eNOS mRNA and protein expressions in mesenteric resistance arteries (M.A.) and aorta of control (open bars) and Bu-Cy treated (filled bars) animals **A**, **E**: Fold change mRNA expression (n = 6) and protein levels (n = 2) of eNOS after Bu-Cy treatment in mesenteric resistance arteries. **C**, **F**: Fold change mRNA expression in aorta (n = 6) and protein levels of eNOS (n = 3) after Bu-CY treatment. Gene expression in panels **A**, **C** is normalized to expression of rS18 gene. Signal intensities of Western blot analyses were normalized to β-actin (panels **E**, **F**). Panel **B** and **D**, show the final product of the RT-PCR that confirms the results of RT-PCR from Bu-Cy treated mice (n = 4) and controls (n = 4). (* = significant values; *P*<0.05).

To examine if the enhanced endothelial relaxant responses in the Bu-Cy resistance arteries could be explained by endothelial nitric oxide synthase (eNOS) expression, we performed mRNA (Figure 4A) and protein expression analyses (Figure 4E) of resistance arteries from the treated and control groups. As shown in Figure 4E, the eNOS protein expression in the resistance arteries of the Bu-Cy treated animals was significantly higher compared to controls (1.59±0.32 (n = 2) and 0.37±0.10 (n = 2), respectively. Moreover, the mean mRNA values were about 1.5-fold higher in the Bu-Cy treated group (n = 6) compared to the control (n = 6) but no significant difference was observed (Figure 4A). We performed the same analysis for the aorta (Figure 4C & F). The result showed significant decrease at the RNA gene expression and slight but not significant increase in eNOS protein. We also evaluated the RT-PCR final product using agarose gel electrophoresis. The final product of the RT-PCR confirmed the increase of the mRNA in the mesenteric arteries from Bu-Cy treated mice (Figure 4B) (average intensity; 268±20 and 166±16 for Bu-Cy and control mice respectively, P<0.01). The results also showed no significant changes in the aorta between the two groups (Figure 4D) (242±6 and 269±13 for Bu-Cy and control mice, respectively P = 0.095).

### Blood pressure recording

**Figure 5:**
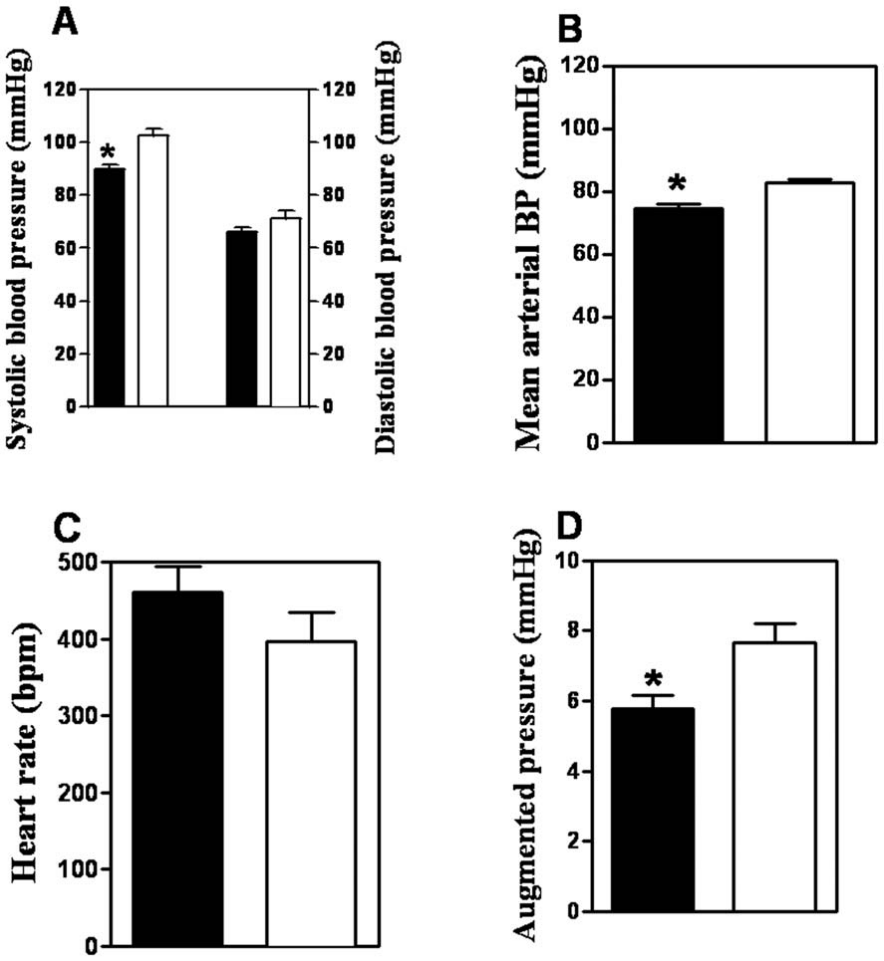
Blood pressure and heart rate in control and Bu-Cy treated animals. Blood pressure and heart rate in control (open bars, n = 3) and Bu-Cy treated (filled bars, n = 5) animals. (**A**) systolic and diastolic pressures (**B**) mean arterial pressure, (**C**) heart rate and (**D**) augmented pressure from pulse wave analysis. (* = significant values; *P*<0.05).

To examine possible *in vivo* cardiovascular effects of the alterations in resistance arterial structure and endothelial signaling we examined blood pressure, heart rate and pulse wave properties in anesthetized animals. As seen in Figure 5, the Bu-Cy treated animals had a significantly lower systolic blood pressure (Figure 5 A), decreased augmented pressure (Figure 5 D), decreased mean arterial blood pressure (Figure 5 B) and increased heart rate (Figure 5 C) compared to controls. In separate experiments we also measured blood hematocrit which was significantly lower in the Bu-Cy treated group (35.0±0.6%, n = 7) compared to the controls (40.3±0.6%, n = 7), *P*<0.0001).

### Transmission electron microscopy (TEM)

**Figure 6:**
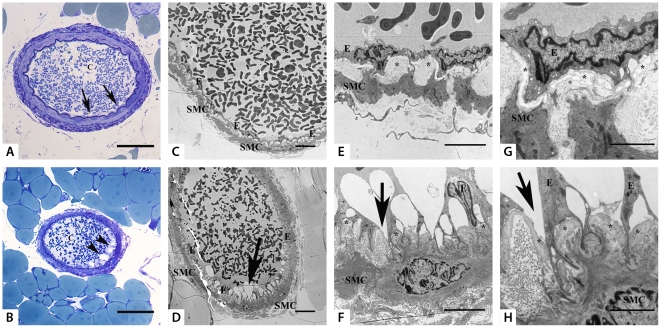
Morphological and ultra-structure images of mesenteric arteries of control and Bu-Cy treated animals. Light microscope examination of semi thin, resin embedded sections stained with toluidine blue O of an arteriole from control mesenteric resistance arteriole showing a uniform endothelial surface (arrows) (A) and an arteriole from Bu-Cy treated mesenteric showing more rounded and frequent blebs at the surface (arrowhead) (B). Bars = 50 µm, C = capillary lumen. Electron microscope examination of: (**C**) Overview of arteriole from control mesentery showing a uniform endothelial cell surface. (**D**) Overview of mesenteric arteriole from treated mouse showing large vacuoles in the lumen at the surface of the vessel wall (arrow) and more rounded endothelial cells in some areas. (**E**, **G**) Capillary cell walls in controls show even and unbroken endothelial cell-cell contact and attachment to the extracellular matrix. (**F**, **H**) Capillary wall from a treated animal show detaching of endothelial cells creating large vacuoles between cell and extracellular matrix. Endothelial cell-cell contacts are disrupted creating gaps (arrow) between cells. E in the Figure = endothelial cell, SMC = Smooth muscular cell, * = elastic fibers. Bars = 10 µm (**C**, **D**), 5 µm (**E**, **F**) and 2 µm (**G**, **H**).

Figure 4 A shows a normal structure of a mesenteric artery of an untreated animal, while Figure 4 B shows an image of an artery of a treated animal with irregular structure of the endothelial cells. The arterioles from the mesenteric arteries of the control group showed that the endothelial cell surface is in close contact with the extracellular matrix and the elastic fibers (Figure 6C). The endothelial cell to cell contacts showed an even and unbroken line (Figure 6E & G). In animals treated with Bu-Cy, the endothelial cells were frequently more rounded and more uneven (Figure 6D). In some areas in the vessel the endothelial cell to cell contact showed a prominent difference compared to the controls. The endothelial cells were detaching from the extracellular matrix including elastic fibers. Furthermore, the endothelial cell to cell contacts were disrupted creating gaps between the cells (Figure 6F & H).

## Discussion

Stem cell transplantation (SCT) is a curative treatment for several malignant and non-malignant disorders. A conditioning regimen is a prerequisite for a successful transplantation and different conditioning regimens have been utilized. Initially, when SCT was introduced, total body irradiation (TBI) as a myeloablative therapy, in combination with Cy as an immunosuppressive therapy, were used as a conditioning regimen. Busulphan was introduced as a substitute for TBI to avoid growth retardation and CNS damage in pediatric patients [39], [40]. Today, a combination of Bu and Cy is commonly used [41]. Although clinical results of SCT are constantly improving, cardiovascular complications of SCT remain relatively uncommon but potentially serious side effects for long-term survivors. Moreover, the mechanisms of an early event in cardiovascular toxicity have not yet been satisfactorily elucidated.

To examine the mechanisms involved in the cardiovascular complications associated with SCT and conditioning regimen, we have used a mouse model for SCT based on Bu-Cy conditioning. The mouse model was first developed in our group to mimic the feature of graft versus host disease (GVHD) after SCT in recipients receiving Bu-Cy conditioning regimen [35]. Moreover, the Bu-Cy treatment conditions and doses are adjusted to simulate the effects of the Bu-Cy regimen used in clinical practice. In this study we examined the effects at about 1 week after the last treatment and our result thus reflects the acute effects of Bu-Cy conditioning.

Bu-Cy treatment has been reported to be involved in cardiac complications, but the role of cyclophosphamide in cardiovascular toxicity has been far better established compared to that of busulphan. Cyclophosphamide has been reported to cause cardiac and vascular toxicities after SCT and after cancer treatment [42], [43], [44], [45]. However, the mechanisms underlying this toxicity are not fully understood. In the present study we investigated the effect of the conditioning regimen on the vascular system. We chose to focus on the Bu-Cy combined regimen because of several factors. First of all, this combination is the most common conditioning regimen used in a clinical setting. Secondly, busulfan is an active drug while cyclophosphamide is a prodrug that has to be activated by the hepatocytes. In vitro studies should include all the active metabolites separately and/or in combination to avoid misleading results. However, it is of great interest to examine the effects of the components and/or their metabolites individually.

Bu-Cy conditioning regimen has been reported to cause tissue injury, which was confirmed in our present study by the observation of damaged endothelial cells. However, this injury could not be caused by inflammation due to the injection. In our previous studies [46], [47], neither inflammatory cells nor elevation in inflammatory cytokines were observed after conditioning. The higher expression of eNOS may be a result of a protective mechanism following tissue injury/inflammation [48], [49], which might be caused by the circulating cytotoxic metabolites of cyclophosphamide (e.g., acrolein, 4-hydroxy-Cy, chloroacetaldehyde). Patel et al recently reported the effect of acrolein on pulmonary artery endothelial cells [50]. We report that the Bu-Cy regimen specifically affected the microarterial vasculature. The resistance sized arterial vessels are considered to contribute to vascular resistance [51]; their structure and mechanical properties significantly influence systemic blood pressure [52], [53]. The localization of the Bu-Cy induced changes to the resistance arteries might thus be an important component in the late vascular system complications and could potentially affect perfusion and function of some organs. Small changes in the vascular diameter have high impact on flow resistance and thereby blood pressure [54]. Our *in vitro* examinations have shown that the resistance arteries of Bu-Cy treated animals had a larger diameter than controls. Since the vessels were obtained from defined vessel segments in the mesenteric vascular tree, we consider the structural changes to reflect a remodeling of vessels rather than a selection of larger vessels. The microvasculature has a significant ability to rapidly adapt to alterations in pressure and/or blood flow [55], [56] on a time scale of days to weeks. It is therefore likely that the Bu-Cy regimen induces a remodeling of small arterial vessels towards an increased lumen as revealed by the larger optimal circumference. We also observed an increased active tension and increased wall stiffness, which suggests a thicker vessel wall with enhanced wall tensions. Since increased blood pressure is associated with increased vascular wall thickness and stiffness in many hypertension models [57], the finding that the lowered blood pressure in the Bu-Cy treatment model is associated with increased wall thickness might suggest that the vessel wall is compensating for the dilatation and effects of LaPlace's law to keep the wall tension constant. This is also consistent with a mechanism where the dilatation, possibly via increased endothelial NO release, discussed below. The changes in structure of the resistance arteries were associated with a significantly enhanced relaxation in response to endothelial stimulation. A combination of a structural change towards a larger lumen and enhanced endothelial relaxation would lower blood flow resistance and blood pressure. The reactivity to the adrenergic contractile agonist was unaltered in the resistance arteries excluding major alterations in the alpha-adrenoceptor contractile signaling in resistance vessels. Our *in vivo* measurements also clearly show a lowered systemic blood pressure, which suggests lowered systemic resistance. Possibly the increased heart rate is a compensatory mechanism for a lower resistance. It should be noted that the hematocrit is lowered after Bu-Cy treatment (present study, [58]), which would further lower flow resistance due to decreased blood viscosity.

We report an upregulation of endothelial nitric oxide synthase (eNOS) in the wall of resistance arteries after Bu-Cy treatment. This is most likely the main mechanism behind the enhanced acetylcholine induced relaxation in the vessels. The link between eNOS expression and blood pressure is well established [59] and the increased eNOS expression and enhanced endothelial responses can constitute a mechanism for the lowered blood pressure after the Bu-Cy regimen. The link between Bu-Cy treatment and altered eNOS expression remains to be examined. It is also possible that the compounds influence the cellular signaling in the vascular endothelium or the production of NO directly. This could be due to damage caused by the Cy metabolites, e.g., acrolein which has been reported to increase NO production [60]. The lowered hematocrit and anemia can be further contributing factors. Erythropoietin can affect eNOS expression in different ways [61], [62] and eNOS expression is increased in animal models of sickle cell anemia [63]. The endothelial function and eNOS expression in the aorta were less affected by the Bu-Cy treatment (*P* = 0.095), which can reflect differences in the endothelial sensitivity between vessel types or in the *in vivo* exposure to the compound. Another explanation can be the earlier /faster transcription and protein synthesis in the aorta compared to the mesenteric artery. It should be noted that NO signaling affects vascular structure [64] and the altered NO signaling can be secondary to the structural injury in the resistance arteries. Recently, Perry et al. have reported that bone marrow-derived endothelial progenitor cells do not restore normal arterial endothelium in young transplanted endothelial nitric-oxide synthase deficient or wild type mice [65]. We provide morphological data after treatment with Bu-Cy and report effects on endothelial cells in the mesenteric arteries with detachment and formation of gaps (Figure 6). Such endothelial gaps may allow for uncontrolled leak of fluids from the lumen of arterioles into the extracellular matrix of the mesenteric arteries resulting in edema. The link between the morphological changes and the increased eNOS expression in correlation with increased endothelium mediated relaxation warrant further investigation. A change in endothelial function and integrity may also be involved in other types of tissue damage such as the hemorrhagic cystitis following cyclophosphamide treatment [26], [27]. Recently, Zeng et al have reported an increased number of circulating endothelial cells during the early phases of conditioning using methotrexate/cyclophosphamide or Bu-Cy as a sign of vascular endothelium injury in a transplantation mouse model [66], [67].

The large elastic artery aorta was not affected in a manner similar to that of resistance arteries. These larger vessels would mainly contribute to pulse wave properties and possibly load on the heart. No structural changes could be detected on the basis of our mechanical experiments and the endothelial function was unaffected. The sensitivity to noradrenalin was increased suggesting that these vessels could have an increased tone and wall tension *in vivo*, particularly during episodes of enhanced sympathetic drive or increased plasma concentrations of catecholamines. This change might have an effect in some physiological or pathophysiological situations with high adrenergic tone, but since we could not detect an increase in blood pressure, a major effect on vascular compliance of the altered adrenergic signaling in resting conditions can be excluded. In the clinical setting, hypertension has been reported after SCT. The mechanism underlying the differential effects of By-Cy on large and small arteries is unknown; it might relate to the bioavailability or metabolism of the drugs in the different vessels.

Our data indicate enhanced endothelial relaxation and alterations of endothelial structure in resistance arteries. These effects appear to be dominant over the enhanced adrenergic responses of the aorta: the net result being a lower blood pressure. Most likely, this reflects an early event in the cardiovascular changes induced by Bu-Cy conditioning. It is possible that longer observation periods, beyond the period focused upon in this study, would reveal the development of hypertension as a result of further changes in endothelial function in resistance arteries as well as a larger impact of the large arterial reactivity changes. Another explanation of the hypertension observed in patients is that patients during SCT are treated with multiple drugs including calcineurin inhibitors that are known for their hypertensive effect.

In the present investigation, using the current regimen and observation period, we could not detect any cardiac weight changes. This may indicate that major alterations in cardiac gross structure or edema formation have not developed. Blood pressure was lower and the heart rate higher in treated animals compared to controls, suggesting an autonomic stimulation of the heart possibly as a result of lowered vascular resistance, as discussed above. However, a longer observation period and examination of cardiac ultrastructure together with functional data are warranted to fully assess possible cardiovascular changes.

In conclusion, we have shown that Bu-Cy treatment has a significant acute impact on the vascular system with selectivity for the smaller resistance arteries controlling blood pressure. After treatment, the vessels become larger and have increased endothelium mediated relaxation, most likely via an increased expression of eNOS. Interestingly, the increased eNOS expression and endothelium mediated relaxation were associated with altered endothelial structure possibly affecting wall permeability. The endothelium performs several functions in the vascular wall. Our analysis of vessel structure suggests that the barrier function can be altered, possibly associated with an increased risk for with extravasation or edema formation in some vascular beds. At the same time, the endothelial relaxation of vascular tone is enhanced, showing that the structural changes do not impair the NO mediated relaxant function, but rather leads to an upregulation. In contrast, structure and endothelial function of larger arteries are less affected, although these vessels have increased reactivity to adrenergic agonists which may contribute to increased wall stiffness, and under high adrenergic tone may result in increased cardiac load.

High doses of alkylating drugs such as cyclophosphamide and ifosfamide may result in reversible heart failure and life-threatening arrhythmias, while the antimetabolites 5-fluorouracil and capecitabine were shown to induce myocardial ischemia. Moreover, anthracyclines such as daunorubicin and doxorubicin were shown to be involved in the development of cardiomyopathy. Introducing Cy to the conditioning regimen prior to SCT increased the onset of cardiotoxicity [28], [29], and a positive correlation between the dose of Cy and severity of cardio toxicity was reported [30]. Unfortunately, the symptoms usually appear 10 to 20 years after SCT in long term survivals [31], but cardiac failure has also been reported within weeks of Cy exposure [32].This is the first study to show that Bu-Cy conditioning prior to SCT causes vascular injury and remodeling, and that it alters reactivity of different vascular segments divergently. These results may contribute to a better understanding of cardiovascular complications reported after SCT in order to enhance prophylactic treatment strategies and/or to optimize conditioning regimen using other drugs.
